# Oral Malignant Melanoma in a Patient With Neurofibromatosis Type 1: An Extremely Rare Association

**DOI:** 10.7759/cureus.25331

**Published:** 2022-05-25

**Authors:** Soufiane Berhili, Mohammed Rezzoug, Ahmed Ben Sghier, Mohammed Moukhlissi, Loubna Mezouar

**Affiliations:** 1 Faculty of Medicine and Pharmacy, Mohammed First University, Oujda, MAR; 2 Departement of Radiation Oncology, Hassan II Oncology Centre, Mohammed VI University Hospital, Oujda, MAR; 3 Department of Radiation Oncology, Hassan II Oncology Centre, Mohammed VI University Hospital, Oujda, MAR; 4 LPMR, Faculty of Sciences, Mohammed Premier University, Oujda, MAR; 5 Faculty of Medicine and Pharmacy, Mohammed Premier University, Oujda, MAR

**Keywords:** rare association, von recklinghausen disease, radiotherapy, neurofibromatosis type 1, oral melanoma

## Abstract

Neurofibromatosis type 1 (NF1) is a genetic disorder associated with high rates of neural crest-derived tumors, both benign and malignant. Many series have identified cutaneous melanoma as a rare tumor among cancers occurring in individuals with NF1 disease, but the mucosal location has to date never been reported. In this paper, we report an oral melanoma occurring in a patient with NF1 disorder, diagnosed at a locally advanced stage, successfully managed by definitive external beam radiotherapy, along with a comprehensive literature review on the melanoma-NF1 association.

## Introduction

Neurofibromatosis type 1 (NF1), also called Von Recklinghausen disease, is an inherited autosomal dominant disorder that occurs approximately once every 3000 live births [1]. It results from mutations in the NF1 gene encoding the Neurofibromin protein: a negative regulator of RAS-dependent growth and differentiation of neural crest-derived tissue [2].

Clinical manifestations of NF1 have been linked to outcomes in all organ systems [3]. Patients with this condition have large phenotypic variability but also higher rates of neural crest-derived tumors than other individuals, both benign and malignant [4-6]. Based on cohort studies, the incidence of cancer in NF1 individuals is approximately fourfold higher than in the general population [4].

The association between NF1 and some tumor types is well recognized, including peripheral nerve sheath tumors (neurofibromas), malignant peripheral neural sheath tumors (MPNSTs), pheochromocytomas, and optic glioma. The lather is even considered one of the diagnostic criteria of NF1 [1,2]. The risk of melanoma in individuals with NF1 conditions is only minimally increased, although melanoma cells frequently host mutations in the NF1 gene. Currently, there remains a lack of evidence for an increased risk of malignant melanoma in patients with NF1 [2,7].

Melanomas with NF1 mutations typically occur on chronically sun-exposed skin [7]. To date, extra-cutaneous melanomas occurring specifically in patients with NF1 disease have never been reported. We report here a case of oral melanoma occurring in a patient with NF1 disorder.

## Case presentation

**Figure 1:**
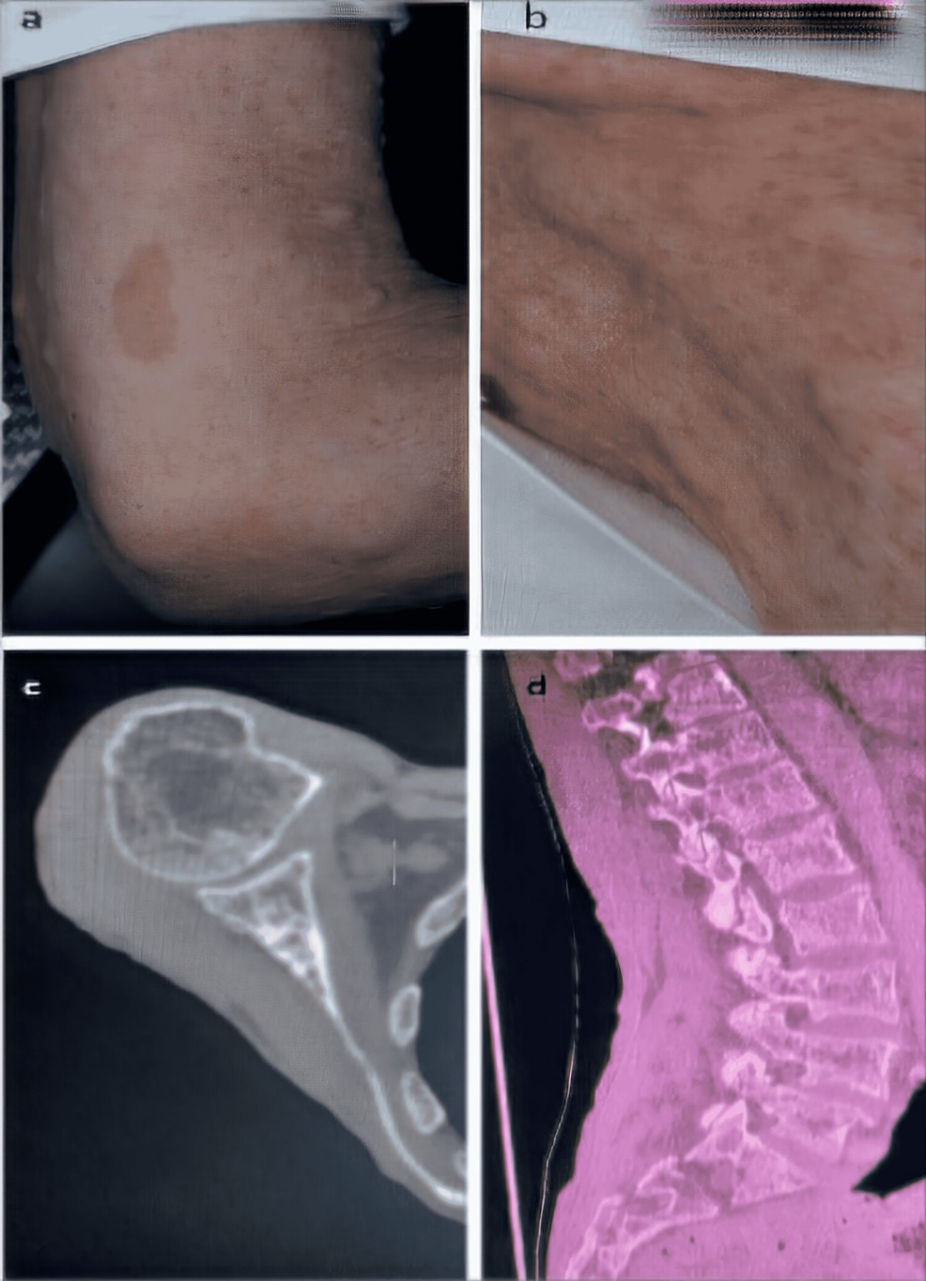
Clinical criteria of the NF1 diagnosis. (a) Multiple diffuse cutaneous neurofibromas with “café-au-lait” skin macules; (b) bilateral axillary freckling; (c) and (d) radiological bone lesions.

A 64-year-old white-skinned female, known to have an NF1 without a family history of melanoma, was referred by an external otolaryngologist to the department of radiation oncology with a hard palate tumor that had been growing for eight months. The diagnosis of NF1 was made almost 40 years ago, based on the presence of multiple diffuse cutaneous neurofibromas, bilateral axillary freckling, "café-au-lait" skin spots, and specific radiological bone lesions (Figure 1).

**Figure 2:**
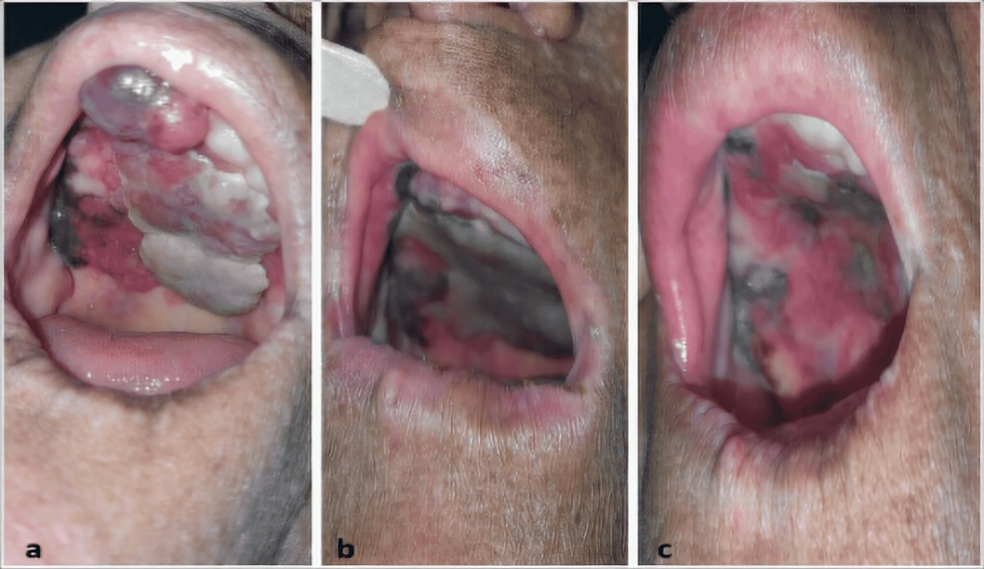
Physical examination finding a multicolored (reddish, yellowish, and blackish) tumor occupying the entire hard palate, extending posteriorly to the soft palate and anteriorly to the gingival and labial soft tissue. (a) Before RT; (b) 1 month after RT; (c) 5 months after RT.

Physical examination at our oncology center found a multi-colored budding tumor of the hard palate, partially reddish, yellowish, and blackish in appearance. The tumor occupied almost the entire arch of the oral cavity, extending posteriorly to the soft palate and anteriorly to the gingival and upper labial soft tissue (Figure 2).

**Figure 3:**
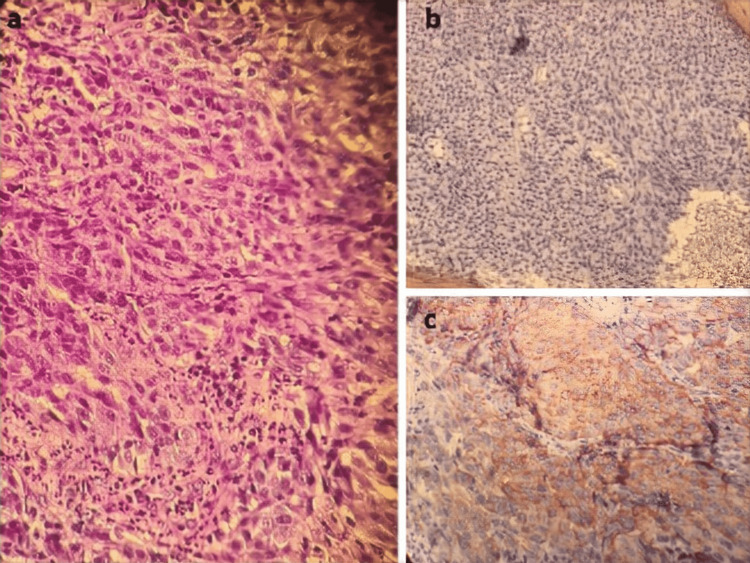
Pathology findings. (a) Examination in HE coloration; (b) and (c) immunohistochemistry findings with positive diffuse HMB-45 and focal PS-100 antibodies staining.

Histopathological examination of the biopsy found an infiltrating malignant trabecular proliferation made of poorly differentiated epithelioid cells, with marked anisocytosis, fine and heterogeneous chromatin, and prominent nucleoli. These cells were surrounded by a polymorphic fibro-inflammatory stroma. An immunohistochemical (IHC) study found positive labeling of the tumor cells with HMB-45 (diffuse staining) and PS-100 (focal staining) antibodies and negative labeling with anti-CK7, anti-CK5/6, anti-P40, anti-AE1/AE3, and anti-CD34 antibodies (Figure 3).

**Figure 4:**
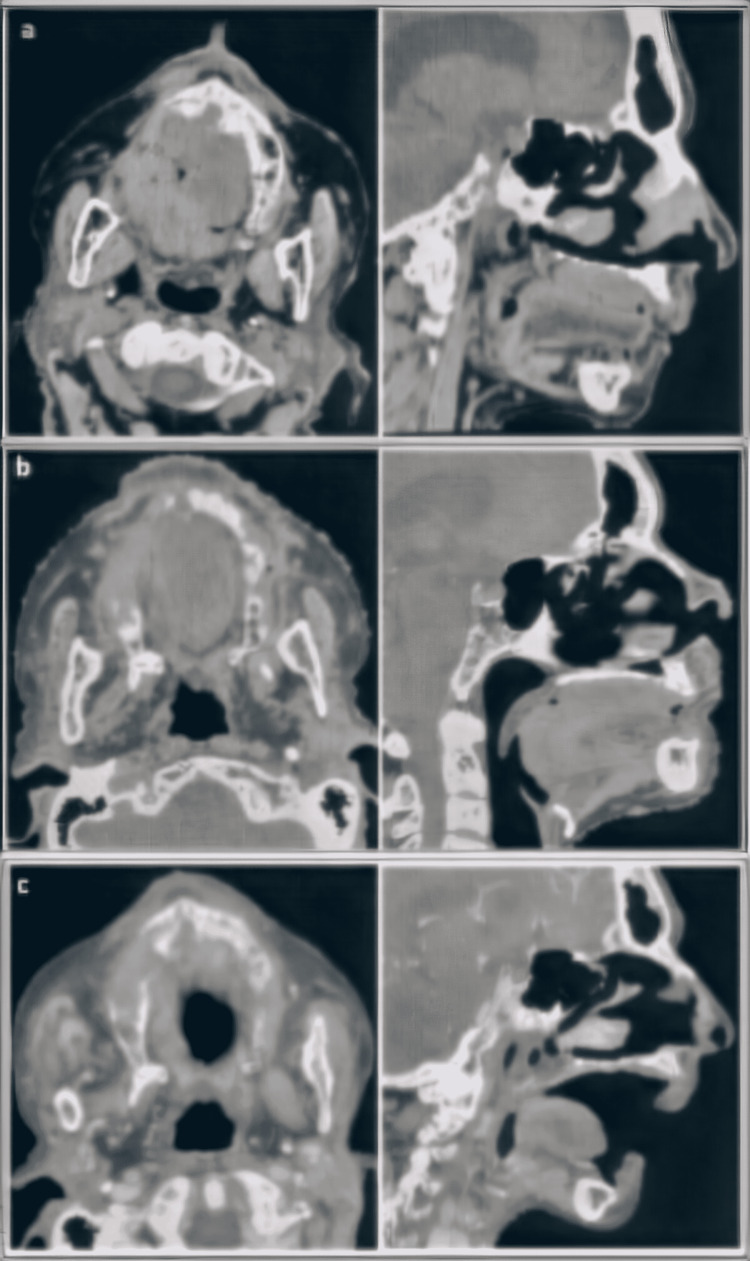
Axial and sagittal computed tomography images showing the hard palate tumor. (a) Before RT; (b) 1 month after RT; (c) 5 months after RT.

A computed tomography (CT) scan showed a tissue lesion of the palate measuring 50 mm × 25 mm in diameter, infiltrating the upper labial soft tissues anteriorly, and the upper jawbone superiorly (Figure 4).

No radiologically suspicious cervical lymph nodes were detected. A thoracic-abdominal-pelvic CT scan found no distant secondary lesion. The patient was referred to the maxillofacial surgery department for resectability assessment, but the local extent of the tumor prevented any appropriate carcinological procedure. Then, based on the advice of the multidisciplinary oncology meeting, it was decided to offer the patient primary definitive external beam radiotherapy and to reserve chemotherapy for any local or distant progression.

**Figure 5:**
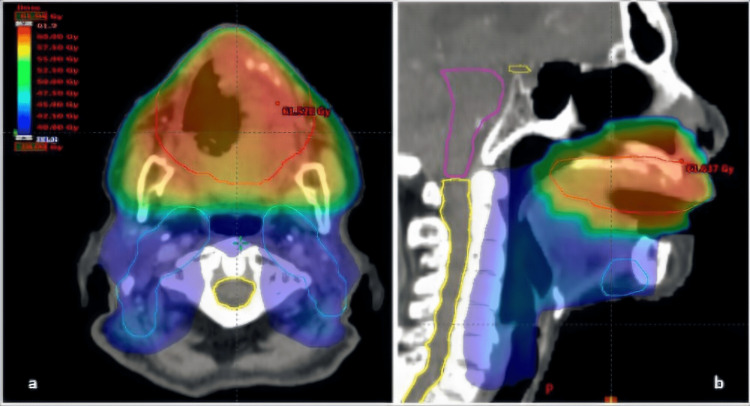
Dosimetric planification of RT, with colorwash dose coverage of the hard palate primary tumor PTV (in red) and the cervical nodal PTV (in blue), in orthogonal planes. (a) Axial plan; (b) sagittal plan.

A total dose of 57 Grays (Gy) in 23 daily fractions (2.5 Gy/fraction, 5 days/7) was delivered to the primary tumor planning target volume (PTV), and 40 Gy in 16 daily fractions (2.5 Gy/fraction, 5 days/7) was delivered to the bilateral cervical nodal levels Ia, Ib, II, and III. We have used 6 Megavolt photon beam radiotherapy in a three-dimension conformal technique (Figure 5).

The clinical tolerance was globally satisfactory; the patient developed a grade 2 acute radiomucitis successfully treated symptomatically. Five months after treatment, the patient was alive with no signs of local or distant tumor recurrence.

## Discussion

The link between cutaneous melanoma and NF1 genetic disorder has been presented in many case reports and case series (Table 1).

**Table 1:** Literature overview of the association between melanoma and NF1, with prevalence rates and tumor locations.

Authors/country/year of publication	Number of NF1 patients	Number of melanomas	Prevalence rate	Location of melanoma
Brasfield et al./USA/1972 [8]	110	6	5.5%	Unspecified
Knight et al./USA/1973 [9]	45	1	2.2%	Cutaneous
Hope et al./Sweden/1981 [10]	395	0	0%	–
Rubenstein et al./USA/1985 [11]	791	4	0.51%	Cutaneous
Sorensen et al./Denmark/1986 [12]	212	1	0.47%	Cutaneous
Zoller et al./Sweden/1997 [4]	70	1	1.4%	Cutaneous
Rasmussen et al./USA/2001 [13]	3,770	12	0.32%	Cutaneous
Guillot et al./France/2004 [14]	671	3	0.45%	Cutaneous
Seminog et al./UK/2013 [6]	6,739	19	0.28%	Cutaneous
Uusitalo et al./Finland/2016 [15]	1,404	3	0.21%	Cutaneous
Zhang et al./USA/2019 [2]	857	1	0.1%	Unspecified
Total (all literature cohorts)	15,064	51	0.34%	–

However, statistical evidence suggesting an increased risk of melanoma in the NF1 population remains controversial [2]. Indeed, cohorts of NF1 patients report heterogeneous prevalence rates of melanoma, ranging from 0% to 5.5%, as listed in Table 1 [4,8-15]. Nevertheless, when the total number of melanoma cases from all series is summed up and compared to the number of NF1 patients, the outgoing calculated prevalence rate of 0.34% is only minimally increased compared to that in the general population.

As shown in Table 1, many series found melanoma to be one of the rare malignancies that could complicate the course of NF1 patients. However, none of the previous publications has reported the occurrence of a mucosal melanoma (MM) in these patients. The present case is the first in the English-written literature reporting the occurrence of a MM during the course of NF1.

MMs are a rare subtype of melanomas, with a proportion ranging between 0.5% and 7.5% of all melanomas [16-19]. They can arise from mucosal melanocytes of the head and neck, vulvar-vaginal, digestive, or urinary tract [17,20]. However, the anatomical site of origin was not found to impact the prognosis of MM in two large series of 700 and 237 patients, respectively [21,22]. These tumors are often associated with a very poor prognosis [16]. Unlike the five-year survival rate of 90% for cutaneous melanoma (all stages combined), MM has a very low rate of below 15% [21,23].

For localized oral melanoma, the only effective treatment with curative intent is complete surgical resection with negative pathological margins [24,25]. However, surgery is not routinely performed for all patients because a complete procedure is often challenging and highly morbid in its oral location. In addition, even after a complete resection with wide margins, local recurrence occurs approximately within the first year after surgery, quickly followed by distant dissemination [26,27]. This unfavorable evolution has led many authors to question the impact of radical resection on survival, given the almost inevitable metastatic progression, emphasizing the importance of maintaining a good quality of life [25,28-31]. For our patient, surgical excision was not planned due to the initial extent of the tumor at diagnosis. Thus, local therapy was based on definitive external beam radiotherapy.

In localized unresectable MM, primary curative radiotherapy is considered the most effective treatment modality, achieving high local control rates of over 70% in recent publications, in contrast to the label of relative "radio-resistance" that has been historically attached to melanoma [16,32,33]. Indeed, the first attempts to irradiate melanomas used RT techniques and devices available at the time (which are currently considered old and obsolete), leading to unsatisfactory outcomes [32,34]. Nowadays, the deeper knowledge of the radiobiological behavior of melanoma cells, along with the progress made in modern planning systems and sophisticated devices, has led to the setting of RT as a cornerstone of melanoma management [32,35].

Radiobiologically, the ability of melanoma cells to repair sub-lethal radio-induced DNA injuries (supported by a low α/β ratio) suggests that high doses per fraction (hypofractionation) would be more effective in causing irreparable DNA damage, and thus achieving the best tumor control [36]. However, despite these solid theoretical arguments, clinical findings on the optimal fractionation schedule for melanoma RT remain controversial. While many retrospective studies found better outcomes with doses per fraction ≥4 Gy, the RTOG 83-05 randomized controlled trial, which compared moderate (2.5 Gy/fraction) versus extreme (8 Gy/fraction) hypofractionation failed to show any clinical benefit for the extreme hypofractionation regimen [32,37]. These findings guided us in our patient's treatment strategy, which consisted of a dose per fraction of 2.5 Gy for a total dose of 57.5 Gy to the primary tumor.

With regard to RT volumes, it is generally accepted that elective nodal irradiation is unlikely to influence the overall course of MM, and the target volume should only include the primary gross tumor in clinically node-negative disease [33,38]. However, given the reported higher nodal failure rates of oral cavity primaries, elective nodal irradiation might be indicated even in N0 disease [39]. Our patient received elective nodal RT to a total dose of 40 Gy in 16 fractions of 2.5 Gy, in an attempt to reduce regional recurrence probability.

RT has also a major role against MM in adjuvant settings where it improves local disease control, although with no benefit on overall survival [39-42]. Also for palliation, RT achieves good tumor debulking rates and improves the quality of life [16].

## Conclusions

In conclusion, this case demonstrated that oral melanomas should be part of the tumors screened in NF1 patients. It also refutes the stigmatized idea of radioresistance of melanomas, illustrating the major role that definitive RT plays in their management. New radiobiological approaches reflecting the good response of mucosal melanomas to radiotherapy are expected.
